# Biases in the metabarcoding of plant pathogens using rust fungi as a model system

**DOI:** 10.1002/mbo3.780

**Published:** 2018-12-25

**Authors:** Andreas Makiola, Ian A. Dickie, Robert J. Holdaway, Jamie R. Wood, Kate H. Orwin, Charles K. Lee, Travis R. Glare

**Affiliations:** ^1^ Agroécologie, AgroSup Dijon, INRA Université Bourgogne, Université Bourgogne Franche‐Comté Dijon France; ^2^ Bio‐Protection Research Centre Lincoln University Lincoln New Zealand; ^3^ Bio‐Protection Research Centre, School of Biological Sciences University of Canterbury New Zealand; ^4^ Manaaki Whenua – Landcare Research Lincoln New Zealand; ^5^ Waikato DNA Sequencing Facility, School of Science University of Waikato Hamilton New Zealand

**Keywords:** cloning, Illumina, Ion Torrent, next‐generation sequencing, plant pathogens, Pucciniales

## Abstract

Plant pathogens such as rust fungi (Pucciniales) are of global economic and ecological importance. This means there is a critical need to reliably and cost‐effectively detect, identify, and monitor these fungi at large scales. We investigated and analyzed the causes of differences between next‐generation sequencing (NGS) metabarcoding approaches and traditional DNA cloning in the detection and quantification of recognized species of rust fungi from environmental samples. We found significant differences between observed and expected numbers of shared rust fungal operational taxonomic units (OTUs) among different methods. However, there was no significant difference in relative abundance of OTUs that all methods were capable of detecting. Differences among the methods were mainly driven by the method's ability to detect specific OTUs, likely caused by mismatches with the NGS metabarcoding primers to some *Puccinia* species. Furthermore, detection ability did not seem to be influenced by differences in sequence lengths among methods, the most appropriate bioinformatic pipeline used for each method, or the ability to detect rare species. Our findings are important to future metabarcoding studies, because they highlight the main sources of difference among methods, and rule out several mechanisms that could drive these differences. Furthermore, strong congruity among three fundamentally different and independent methods demonstrates the promising potential of NGS metabarcoding for tracking important taxa such as rust fungi from within larger NGS metabarcoding communities. Our results support the use of NGS metabarcoding for the large‐scale detection and quantification of rust fungi, but not for confirming the absence of species.

## 1 INTRODUCTION

Plant pathogens are a critical threat to global food security (Bebber & Gurr, 2015), the conservation of natural ecosystems, and the future resilience and sustainability of ecosystem services (Bever, Mangan, & Alexander, 2015). Because of their importance, there is a huge interest to biomonitor plant pathogens cost‐effectively at large scales without the need of culturing and before possible disease outbreaks.

Rust fungi (Pucciniales) constitute one of the largest groups of plant pathogens, with about 7,800 described species (Helfer, 2014), and some rust species can have large economic and ecological impacts. For example, myrtle rust (*Austropuccinia psidii*) is currently decimating a wide range of Myrtaceae around the world (Fernandez Winzer, Carnegie, Pegg, & Leishman, 2018; Glen, Alfenas, Zauza, Wingfield, & Mohammed, 2007), such as the endemic *Eugenia koolauensis* in Hawai‘i and the endemic *Rhodamnia rubescens* in native forests in Australia (Carnegie et al., 2016). Coffee leaf rust (*Hemileia vastatrix*) is substantially damaging Coffee plantations worldwide (Talhinhas et al., 2017). Similarly, wheat leaf rusts like *Puccinia triticina*, *Puccinia recondite*, and *Puccinia striiformis* are causing serious production losses for one of the world's biggest food crops (McCallum, Hiebert, Huerta‐Espino, & Cloutier, 2012).

While many studies focus on rust fungi as perceived pests, they actually constitute a vital component of natural ecosystem functioning. In contrast to agroecosystems, rusts in their natural ecosystems are less well studied, and some species are threatened by extinction due to global change (Helfer, 2014). Because of the economic and ecological importance of plant pathogens, such as rust fungi, new, reliable, and cost‐effective tools are urgently needed to monitor them at large scales.

Next‐generation sequencing metabarcoding has the potential to develop into an effective method for the molecular identification of multiple plant pathogens from environmental samples (Merges, Bálint, Schmitt, Böhning‐Gaese, & Neuschulz, 2018; Taberlet, Coissac, Hajibabaei, & Rieseberg, 2012). DNA metabarcoding seems especially promising for the monitoring of potential plant pathogens (hereafter pathogens), because it bypasses the need for cultivation and isolation of species, and is able to detect plant pathogenic species when they occur asymptomatically (Malcolm, Kuldau, Gugino, & Jiménez‐Gasco, 2013; Stergiopoulos & Gordon, 2014) or at barely discernible levels. While DNA metabarcoding holds great potential for detecting and monitoring fungi in their environment (Durand et al., 2017; Miller, Hopkins, Inward, & Vogler, 2016; Schmidt et al., 2013), it has not yet been widely applied to pathogens specifically (Abdelfattah, Nicosia, Cacciola, Droby, & Schena, 2015; Merges et al., 2018). It is therefore crucial to more fully understand the potential limitations of this new approach.

Two limitations that frequently arise in NGS metabarcoding studies are the ability to quantify the abundances of different taxa (Deiner et al., 2017; Elbrecht & Leese, 2015), and the introduction of false positives/negatives by PCR amplification, library preparation, and sequencing (Coissac, Riaz, & Puillandre, 2012). Here, we address these two possible limitations of NGS metabarcoding using the group of rust fungi as a model system. We investigate possible differences between NGS metabarcoding and more traditional cloning approaches in the detection and abundance of rust fungal species. We also investigate what causes these differences. We use two primer pairs because our objective in this study was to compare methods using the best available and most appropriate approaches for each method. For the NGS metabarcoding approach, we use two fundamentally different sequencing technologies (Illumina MiSeq and Ion Torrent PGM) and fungal NGS metabarcoding primers to detect rust fungi from within a larger fungal community. We compare these results to a cloning approach, targeting the same gene region but focusing cloning on rust fungi using a rust fungi‐specific primer pair.

We hypothesize that the three methods (Illumina, Ion Torrent, and cloning):

1. differ in their detection of rust species (i.e., observed from expected number of detected rust species)
2. differ in their ability to quantify relative abundances of rust fungal species.

If one or both of the hypotheses are supported, we would then test hypotheses for the mechanisms driving differences among methods. Specifically, we hypothesize that differences among methods are due to:

1. differences in sequence lengths among methods
2. differences in the most appropriate bioinformatic pipelines for each method
3. taxonomic biases of the methods
4. different abilities of methods to detect rare species.

## 2 METHODS

### 2.1 Study site and sampling

We sampled thirty 20 × 20 m grassland plots. All plots were based on an 8 × 8 km grid that is used extensively for national biodiversity monitoring in New Zealand (Allen, Bellingham, & Wiser, 2003) and positioned following the standard protocol of Hurst and Allen (2007). The plots were selected based on the output of the geographic information system and stratified random sampling (Figure S1). We limited our sampling to grassland plots located at altitudes <1,000 m. All sampling was carried out under dry weather conditions between November 2014 and March 2015.

At each plot, samples were collected using a sterilized leaf puncher within 64 min (4 min for each of sixteen 5 × 5 m subplots) to ensure balanced sampling of the whole plot. Every identifiable plant part (e.g., healthy leaves, leaves with lesions, bryophytes, grass stems, lichens, bark, seeds), including healthy as well as diseased plant material, was sampled to get all variants and to maximize rust fungal diversity. Since most of these samples represent above‐ground herbaceous material (mainly leaves), we hereafter refer to these samples simply as “leaf samples.” The leaf samples were immediately pooled by plot, stored in a 50‐ml Falcon tube containing sterilized DMSO‐NaCl solution (20% DMSO, 0.25 M disodium‐EDTA, and NaCl to saturation, pH 7.5), sealed with Parafilm M, and kept at 4°C until laboratory processing.

### 2.2 DNA extraction

The DNA extraction from the pooled leaf samples of each plot was carried out using the Macherey‐Nagel NucleoSpin 96 Plant II kit (robot extraction) following the manufacturer's protocol. We used both provided lysis buffers separately (cetrimonium bromide [CTAB] lysis buffer PL1 and a sodium dodecyl sulfate [SDS]‐based lysis buffer PL2) to enhance the amount of extracted DNA. Five microliters of product was quantified using a Qubit 2.0 fluorometer (Life Technologies) and the broad‐range assay kit following the manufacturer's protocol before equally pooling the extracts from the same plot.

### 2.3 Preparation of next‐generation sequencing libraries

We prepared NGS libraries in a one‐step PCR (Immolase MoTASP protocol) to avoid the risk of contamination, following Clarke, Czechowski, Soubrier, Stevens, and Cooper (2014). We used the fungal primers fITS7: GTGARTCATCGAATCTTTG (Ihrmark et al., 2012) and ITS4: TCCTCCGCTTATTGATATGC (White, Bruns, Lee, & Taylor, 1990), amplifying the highly variable internal transcribed spacer region 2 (ITS2) with universal linker sequences at the 5' end for fITS7: TCGTCGGCAGCGTC and for ITS4: GTCTCGTGGGCTCGG. Illumina adapter sequences with index sequences and complementary linker sequences were as follows:

F: AATGATACGGCGACCACCGAGATCTACAG‐8nt index‐TCGTCGGCAGCGTC,.

R: CAAGCAGAAGACGGCATACGAGAT‐8nt index‐GTCTCGTGGGCTCGG. Ion Torrent adapter sequences with index sequences and barcode adapter sequences were as follows:

F: CCATCTCATCCCTGCGTGTCTCCGACTCAG‐10nt index‐GAT,.

R: CCACTACGCCTCCGCTTTCCTCTCTATGGGCAGTCGGTGAT

The universal fITS7 primer has been noted to exclude certain Ascomycota (*Penicillium*, Orbiliales) and most Mucorales (Ihrmark et al., 2012), but was chosen because it is more fungi‐specific compared to other universal primers (e.g., fITS9 or gITS7, which match some plants because they are degenerated at two positions, potentially overwhelming any fungal signal in leaf substrates). Moreover, the primer pair fITS7 and ITS4 is believed to capture most of the Basidiomycetes, including rust fungi, and its amplicon lengths are well suited to next‐generation sequencing (average of 258.5 ± 27.3 bp for Ascomycota and 309.8 ± 35.6 bp for Basidiomycota) (Bokulich & Mills, 2013; Ihrmark et al., 2012). Purification and size selection (280–520 bp) were performed using a PippenPrep system to exclude primer dimers and high molecular weight DNA, before paired‐end sequencing the samples with the Illumina MiSeq platform (250 cycle PE) at the Australian Genome Research Facility Ltd, Melbourne, Australia, and with the Ion Torrent PGM platform (400 bp SE) at the Waikato DNA Sequencing Facility, University of Waikato, Hamilton, New Zealand.

### 2.4 Preparation of clone libraries

The use of a rust fungi‐specific primer was necessary to focus the cloning procedure on Pucciniales and to get to species resolution. We amplified an approximately 1,400‐bp target region with the rust fungi‐specific forward primer Rust2inv:

GATGAAGAACACAGTGAAA (Aime, 2006) and reverse primer LR6: CGCCAGTTCTGCTTACC (Vilgalys & Hester, 1990), starting in the 5.8S subunit and spanning the highly variable ITS2 region and the three most divergent domains (D1, D2, D3) of the large subunit (LSU, 28S). We performed PCRs for the two DNA extracts of each plot using the TaKaRa Ex Taq DNA polymerase kit (25 µl reaction volumes, containing 2.5 µl 10X Ex Taq buffer, 2 µl dNTP mixture (2.5 mM each), 5 µl 10 µg/ml rabbit serum albumin (RSA), 0.6 µl 10 µM of each upstream and downstream primer, 0.125 µl TaKaRa Ex Taq, 1 µl DNA template, and 13.175 µl of sterilized distilled water). PCR conditions consisted of an initial denaturation step of 2 min at 94°C, 35 cycles of 30 s at 94°C, 1 min at 57°C, and 1.5 min at 72°C, and a final extension of 7 min at 72°C, as initially described by Aime (2006) but using fewer cycles. We pooled 1 µl of PCR product originating from the CTAB and 1 µl from the SDS‐based lysis buffer DNA extractions per plot, and cloned using the Strataclone PCR cloning kit (Agilent, Stratagene), following the manufacturer's protocol, with blue‐white screening of colonies. We conducted a preliminary restriction fragment length polymorphism (RFLP) to determine sufficient sampling depth. The rarest pattern observed occurred five times out of 100 colonies within a plot. On that basis, we picked 50 colonies per plot (1,500 overall), resulting in a 91.47% probability of detecting the rarest OTU. We performed colony PCRs with the plasmid‐specific primer pair M13–20: GTAAAACGACGGCCAG and M13RSP: CAGGAAACAGCTATGACCAT (Wood et al., 2012), using the TaKaRa Ex Taq DNA polymerase kit (15 µl reaction volumes, containing 1.5 µl 10X Ex Taq buffer, 1.2 µl dNTP mixture (2.5 mM each), 0.6 µl 10 µg/ml rabbit serum albumin (RSA), 0.24 µl 10 µM of each upstream and downstream primer, 0.075 µl TaKaRa Ex Taq, colony DNA template, and 10.15 µl of sterilized distilled water). PCR conditions consisted of an initial denaturation step of 12 min at 94°C, 35 cycles of 20 s at 94°C, 10 s at 55°C and 1.5 min at 65°C, and a final extension of 10 min at 65°C, following the method of Wood et al. (2012) but doubling the annealing time at 65°C. After a gel visualization, sequencing of colony PCR products in the forward direction was conducted with the Rust2inv primer at the Bio‐Protection sequencing facility, Lincoln University, New Zealand. Reverse sequencing was not conducted because the gene regions of interest (ITS2, D1, D2, D3) lie within the first 750 bp of the forward sequencing read.

### 2.5 Bioinformatics

We trimmed low‐quality bases at the clone library sequence beginnings and ends, and removed primer and vector sequences. We aligned the sequences using the MUSCLE version 3.8.31 algorithm (Edgar, 2004) and trimmed the beginning, so they start at the same point of the gene region as the sequences from Ion Torrent and Illumina using the fITS7 primer. Identical sequences were de‐replicated and N‐padded to the same length. N‐padding (i.e., adding Ns, which represent any nucleotide) to the end of each sequence until they have the same lengths was needed because the clustering algorithm used considers terminal gaps to be absolute differences. However, N‐padding only was necessary for two short clone sequences. Not N‐padding of these two sequences would have resulted in two additional OTUs but would not have changed the overall results. We clustered the sequences to a 97% similarity threshold without using singletons using UPARSE algorithm (Edgar, 2010). This threshold represents the ITS barcode gap for the overwhelming majority of fungal species, including the subdivision Pucciniomycotina (Schoch et al., 2012).

The forward and reverse Illumina reads were merged using the fastq_mergepairs command of USEARCH version 9.0.2132, and sequences with more than one expected error and less than 175 bp were removed. Ion Torrent sequences were only used if the forward and the reverse primer complement could be found within the sequence and if the sequence was at least 175 bp long. We discarded Ion Torrent sequences with more than two expected errors (EE). We set a higher EE threshold because the mean expected error rate of the Ion Torrent runs at the sequence length of 300 bp was two. We trimmed non‐biological (primer) sequences, allowing 10% bp mismatch using the Python tool “cutadapt” version 1.13 (Martin, 2011) if the forward primer or the reverse primer complement could be found at the sequence ends. Identical sequences were de‐replicated. Illumina and Ion Torrent data were independently clustered to 97% similarity threshold without using singletons, using the UPARSE greedy clustering algorithm (Edgar, 2013).

We constructed a reference database from UNITE and INSD (accessed 20.11.2016) and matched the representative sequence of each OTU to this database using BLAST version 2.5.0+ (Altschul et al., 1997). We considered an OTU to represent the order Pucciniales if it matched Pucciniales sequences in the database >80% identity over at least 150 bp (Nguyen et al., 2016; Schoch et al., 2012). Extraction blanks, and positive and negative controls, were checked for contamination. Tag jumping (false combinations of tags and samples, which cause incorrect assignment of sequences) (Schnell, Bohmann, & Gilbert, 2015) was accounted for by using a regression of the abundance of contaminants versus the maximum of total abundances in all other samples. The coefficient estimate for the 90th quantile regression was then used to subtract that many sequences from all OTUs. Hence, this tag‐jumping correction takes into account the fact that more abundant OTUs are more likely to do tag jumping. We blasted OTUs obtained from the three different methods against each other and considered them to be the same OTU if they matched at >98.5% similarity, which corresponds to approximately 3% clustering of the NGS data using the distance‐based greedy clustering UPARSE algorithm (Edgar, 2013), but allows different sequence lengths as opposed to matching with USEARCH version 9.0.2132 (Altschul et al., 1997; Edgar, 2010, 2013).

### 2.6 Statistical analyses

We used R version 3.4.1 (R Core Team, 2017) for conducting analyses and creating graphs if not stated otherwise. To test whether a method detected more or fewer shared/unique rust fungal OTUs than expected by chance, we used the “permatswap” function of the R package “vegan” version 2.0–7 (Oksanen et al., 2017) to create a null expectation. The simulated community matrices are based on Monte Carlo iterations, whereby the total number of OTUs per plot and total abundance within OTU were kept constant. We tested for differences in OTU abundances among methods using a generalized additive model (GAM) of the package “mgcv” version 1.8–18 (Wood, 2001). A GAM was selected because: (a) it allows beta distribution for the response variable, which in this case was the appropriate distribution for the proportional abundance of each OTU found within a plot (to account for different sequencing depths); and (b) the approach allows testing for OTU and plot as random effects, and interaction between method and OTU. Data were rescaled to exclude zeros and ones, as suggested by Smithson and Verkuilen (2006). Wald test was used to test the significance of each parametric and smooth term (Wood, 2012). To see whether perceived rust fungi communities differ among methods, we converted the obtained community data into Jaccard distance matrices using Wisconsin double standardization. Four plots with zero OTUs, as well as unique communities, had to be discarded because of a dissimilarity of one. We displayed the dissimilarities with nonlinear multidimensional scaling and tested for significance between the configurations using Procrustes rotation and the “protest” function part of the “vegan” package, and the “mantel.test” function of the “ape” package (Paradis, Claude, & Strimmer, 2004). We tested whether a bias among methods was caused by different sequence lengths or bioinformatic pipelines, applying the same sequence length (248 bp) and/or an identical bioinformatic pipeline to all methods. To look for a taxonomic bias in detecting the different methods, we constructed a neighbor‐net phylogeny (Bryant & Moulton, 2004) using Splitstree 4.0 (Huson, Kloepper, & Bryant, 2008) and used chi‐square test to test whether taxonomic clusters are independent of methods. We tested whether a possible difference is due to the detection of rare and dominant OTUs by rerunning all tests using the top and lower 50% of the rank abundance of each method. Species identities are based on the best BLAST match and were displayed as networks using the “igraph” package version 1.0.1 (Csardi & Nepusz, 2006) with edge width representing relative species abundance within method.

## 3 RESULTS

### 3.1 Differences among methods in detection of OTUs

**Figure 1:**
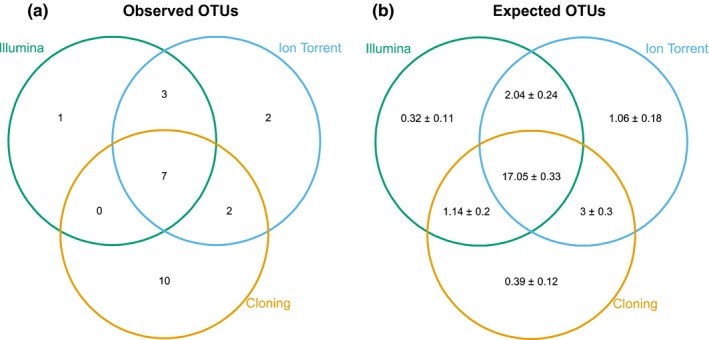
(a) Observed and (b) expected number of rust fungal operational taxonomic units (OTUs) per method. OTUs were considered to be identical among methods when >98.5% BLAST similarity. Expectations were based on Monte Carlo random sampling (100 iterations) and displayed with 95% confidence intervals

There were seven rust fungal OTUs shared among the three methods, which was much less than would be expected by random sampling (17.05 ± 0.33). The difference was driven by OTUs uniquely detected by single methods (Figure 1), that is, Illumina (one unique OTU) and Ion Torrent (two unique OTUs), and cloning (10 unique OTUs). The three methods (i.e., cloning, Illumina, and Ion Torrent) hence differed in detection of rust fungal OTUs.

### 3.2 No differences among methods in relative abundances of shared OTUs and in perceived community composition

**Figure 2:**
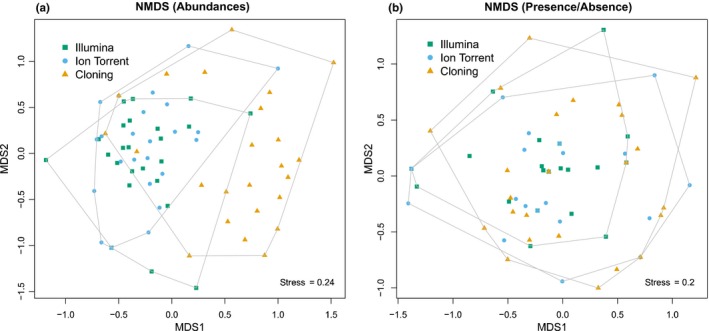
Multidimensional scaling of rust communities (using abundance and presence/absence data) as perceived by three different methods: Illumina (green, squares), Ion Torrent (blue, circles), cloning (orange, triangles). Four plots were dropped because of lack of any detected rust communities in these plots

There was no evidence of differences in quantification of relative abundances among the three methods (i.e., cloning, Illumina, and Ion Torrent) among OTUs that all methods were capable of detecting. A likelihood ratio test between models with and without an interaction term (method × OTU) was not significant (*χ* ^2 ^= 7.62, *df* = 12, *p* = 0.81). In general, rust communities perceived by the three methods did not result in largely different community patterns, as visualized by the overlap of the communities in NMDS (Figure 2). Mantel test and Procrustes analysis confirmed similarity (*p < *0.05) for Ion Torrent/cloning (abundance data), and Ion Torrent/cloning and Illumina/Ion Torrent (presence/absence data).

### 3.3 Mechanisms driving OTU detection differences among methods

**Figure 3:**
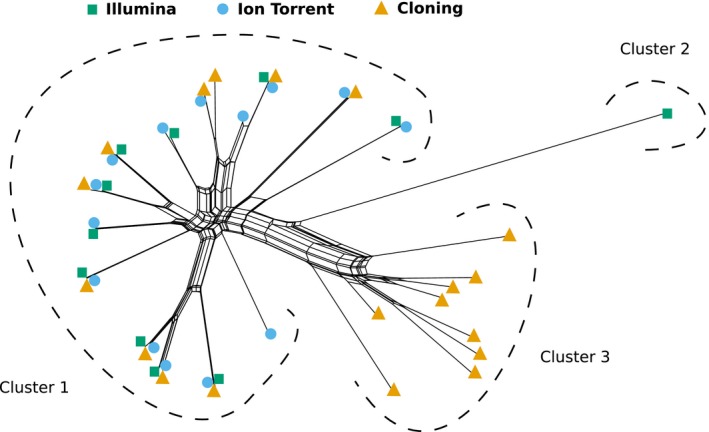
Neighbor‐net phylogeny of rust fungal operational taxonomic units (OTUs) detected by the different methods: Illumina (squares), Ion Torrent (circles), cloning (triangles)

Differences in detection among methods seemed not to be due to sequence length differences among methods. After trimming all sequences to the same length (248 bp), which is the shortest common sequence of all methods, and rerunning the analysis, the number of observed (seven) compared to randomly expected (17) shared rust OTUs stayed unchanged. Differences in detection among methods also seemed not to be due to differences in the most appropriate bioinformatic pipelines for each method. Using an identical bioinformatic pipeline for all methods made differences even more extreme, with only four OTUs shared among methods, compared to seven (with the most appropriate pipelines) or 17 (expected). Differences in detection among methods were due to a taxonomic bias of the methods. Neighbor‐net phylogeny (Figure 3) indicates three taxonomic clusters. Cluster 1 could equally be detected by all methods; cluster 2 was only detected using Illumina; cluster 3 was only detected using cloning. The chi‐square test for independence was significant (*χ* ^2 ^= 17.536, *df* = 4, *p* < 0.01) and confirmed that clusters were not equally formed by the different methods.

**Figure 4:**
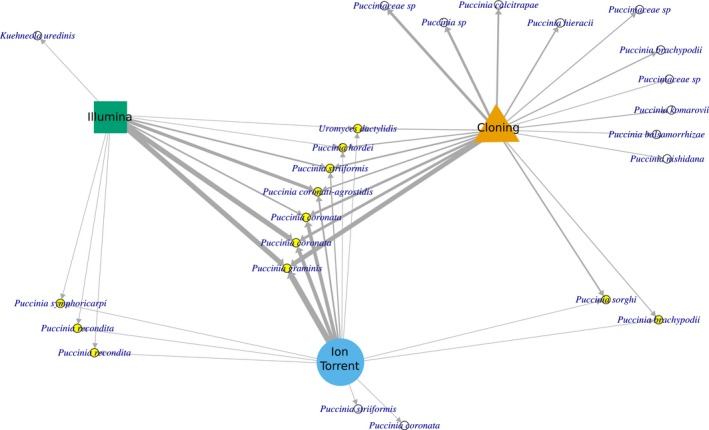
Network representing shared and unique rust fungal operational taxonomic units (OTUs) among methods. Edge width represents proportional abundance of an OTU within method. Species identities are based on their best BLAST match. OTUs found in each method are considered to be identical when showing >98.5% sequence similarity

Species identities of cluster 3 (i.e., uniquely detected by cloning) and cluster 2 (i.e., uniquely detected by Illumina) were displayed in a co‐occurrence network (Figure 4). While Illumina's uniquely detected species is from the genus *Kuehneola*, uniquely detected species from cloning and Ion Torrent are from the genus *Puccinia*. The taxonomic bias seemed not to be driven by poor detection of rare OTUs. The same clusters occur when only considering the upper 50% of rank abundance, hereafter called dominant OTUs (Figures S2 and S3), and when only considering the lower 50% of rank abundance, hereafter rare OTUs (Figures S4 and S5). The number of observed shared dominant (six) and rare (two) OTUs still differs significantly from randomly expected (11.08 ± 0.36 OTUs) shared rust OTUs. This difference in observed from expected is still mainly due to the uniquely detected OTUs from cloning (cluster 2 of Figure S2 and cluster 3 of Figure S4).

Differences in detection among methods seemed to be caused by base pair mismatches of the NGS metabarcoding primer pair. Table 1 shows selected species that were detected by cloning but not by NGS metabarcoding and had at least one base pair mismatch to the NGS metabarcoding primers.

**Table 1:** Metabarcoding primer mismatches to selected species that were detected by cloning but not by metabarcoding

Species	5'‐fITS7 (forward primer) GTGARTCATCGAATCTTTG	3'‐ITS4 (reverse primer) GCATATCAATAAGCGGAGGA
*Puccinia calcitrapae* a	GTGAATCAT**T**GAATCTTTG	GCATATCAATAAGC**A**GAGGA
*Puccinia nishidana* b	....ATCAT**T**GAATCTTTG	GCATATCAATAAGC**A**GAGGA
*Puccinia balsamorrhizae* c	......CAT**T**GAATCTTTG	GCATATCAATAAGC**A**GAGGA
*Puccinia komarovii* d	GTGAATCAT**T**GAATCTTTG	GCATATCAATAAGC**A**GAGGA
*Puccinia hieracii* e	......CATCGAATCTTTG	GCATATCAATAAGC**A**GAGGA

Note

Mismatches are highlighted (bold and underlined).

Sequences were selected from the National Center for Biotechnology Information (NCBI) to cover the gene region of cloning and metabarcoding primers when possible.

Dot indicates no entry of base pair in the database.

Accession numbers are given as footnotes. Accession numbers: ^a^JN204183.1 ^b^HM022141.1 ^c^JN204182.1 ^d^HQ317515.1

## 4 DISCUSSION

This study demonstrates that NGS metabarcoding is an effective technique for large‐scale detection of rust fungus plant pathogens, but that taxonomic biases due to primer selection are a potential limitation. To the best of our knowledge, this is the first study with a real‐world application and comparison of cloning and NGS metabarcoding to survey Pucciniales. We found differences in the detection of rust fungus species among Illumina and Ion Torrent platforms, and cloning followed by Sanger sequencing. However, we found no significant difference in the relative abundances of the rust fungus species that all methods were capable of detecting. The mechanism driving detection differences among methods seemed to be due to a taxonomic bias, which was very likely caused by base pair mismatches of the NGS metabarcoding primer pair to some *Puccinia* species. Otherwise, the consistency among fundamentally different and independent molecular methods shows that NGS metabarcoding and cloning are on a par. Altogether, the results support the application of NGS metabarcoding for the large‐scale detection of plant pathogens (presences) and contradict its application for inferring absence of species, depending on the primer pairs. These findings are important to future metabarcoding studies because they highlight the main source of difference among methods and rule out several mechanisms that could drive differences.

The main difference between the methods (NGS metabarcoding and cloning) was due to their biases in species detection, not quantification. This suggests that previous problems when using quantitative next‐generation sequencing data (Elbrecht & Leese, 2015; Piñol, Mir, Gomez‐Polo, & Agustí, 2015) were probably induced by PCR, and not by the method or sequencing platform per se. Furthermore, this is in line with the finding that the difference in detection between NGS metabarcoding and cloning shows a taxonomic bias. Both the NGS metabarcoding and the cloning primers have either a perfect match or only a maximum of two base pair mismatches to all detected rust fungi in this study. Moreover, the NGS metabarcoding primers were thought to capture most of the Basidiomycetes (Ihrmark et al., 2012; White et al., 1990), including rust fungi. Consequently, the NGS metabarcoding and the cloning primers would be expected to detect a similar assemblage of rust fungi. However, the base pair mismatches of the NGS metabarcoding primer occur in species that are only detected by cloning, and the cloning primer had no mismatches in these species. The lower specificity of the “universal” NGS metabarcoding primers is therefore more likely to discriminate against the amplification of those species when exposed to 100% matching other fungal sequence templates (Bellemain et al., 2010). Lowering the annealing temperatures might help remedy these mismatch biases for this group in the future, particularly as none are very close to the 3' end of primers (Table 1). Although taxonomically clustered, the *Puccinia* species with the base pair mismatch of the NGS metabarcoding primer seemed not to fall into a known taxonomic cluster, like a subgenus (Van der Merwe, Ericson, Walker, Thrall, & Burdon, 2007).

Numerous NGS metabarcoding studies have pointed out that NGS metabarcoding primers can discriminate against certain taxa (Bellemain et al., 2010; Clarke et al., 2014; Elbrecht & Leese, 2015; Schmidt et al., 2013). Some studies have tried to limit this bias to some extent by using quantitative PCR and correction factors (Thomas, Deagle, Eveson, Harsch, & Trites, 2016), primer mixes (Tedersoo et al., 2015), or blocking oligonucleotides to non‐target DNA (Piñol et al., 2015). Ficetola et al. (2010) proposed an “electronic PCR” application to measure barcode coverage and specificity. This in silico approach has proven useful to identify the appropriate barcode gene regions and when comparing different primers for fungi (Bellemain et al., 2010) and vertebrates (Valentini et al., 2016). The results from this study and from the literature, taken together, highlight the importance of primer choice for NGS metabarcoding studies. NGS metabarcoding studies should therefore carefully examine in silico what taxa their primers might discriminate against in order to select appropriate NGS metabarcoding markers and aid the interpretation of results.

This study also ruled out several mechanisms that could possibly drive detection differences between NGS metabarcoding and cloning. We found no evidence that sequence length, most appropriate bioinformatic pipeline, or ability to detect rare species caused any differences among methods. We found that shortening all sequences to the length of the shortest sequence (248 bp) did not change the interpretation of the overall results and resulting phylogeny. Min and Hickey (2007) and Han et al. (2013) showed that reducing sequence length can have effects on the accuracy of phylogenies when DNA barcoding fungi. They also showed that despite some loss of phylogenetic signal, shorter sequences can still resolve the terminal nodes of the phylogeny quite efficiently in most cases. Current next‐generation sequencing technologies still require the amplification of short sequences, and some barcode regions (e.g., the ITS region for fungi) can lack the necessary resolution for particular fungal taxa (Gazis, Rehner & Chaverri, 2011). Despite these challenges, short sequences provide enough resolution at a genus and often a within‐genus level for the majority of fungi (Blaalid et al., 2013). While short sequences have been repeatedly shown to be sufficient for genus‐ or even species‐level identifications (Blaalid et al., 2013; Bokulich & Mills, 2013), future next‐generation sequencing technologies should be able to overcome the current length limitations and provide the field of NGS metabarcoding with even better species delimitations (Goodwin, McPherson, & McCombie, 2016).

Bioinformatic pipelines can have profound effects on the outcome of NGS metabarcoding studies (Flynn, Brown, Chain, MacIsaac, & Cristescu, 2015). In this study, the error rate strongly differed between Illumina, Ion Torrent, and Sanger sequencing runs. Using an identical bioinformatic pipeline, such as identical quality filtering and clustering, resulted in a much lower number of shared OTUs among the methods. These results justify using the most appropriate bioinformatic pipeline for each method. Moreover, we did not find any effect of rare species on detection ability among methods. The same taxonomic bias among the methods occurred when only looking at the dominant or only looking at the rare OTUs. Rare OTUs in NGS metabarcoding data are generally more prone to errors due to the accumulation of errors (Dickie, 2010), tag jumping (Schnell et al., 2015), chimera formation (Edgar, Haas, Clemente, Quince, & Knight, 2011), or false positive/negatives (Ficetola et al., 2010). However, previous studies have shown that if these problems associated with rare OTUs are overcome, the ability of NGS metabarcoding to detect rare species is equal to or exceeds non‐molecular methods (Valentini et al., 2016; Zhan et al., 2013).

Next‐generation sequencing metabarcoding seems appropriate for the large‐scale detection of rust fungi and less appropriate for inferring absence of species. For example, the species *Puccinia sorghi* was initially present in the raw data of all three methods. However, only two sequences of this species were present in the Illumina raw data. These two sequences exhibited a point mutation or a possible sequencing error in their reverse sequence read and got treated as unique sequences (singletons) after merging. Hence, although initially present in the Illumina raw data, these two sequences could not form an OTU. This phenomenon of species getting lost during merging of paired‐end sequencing has been noted earlier by Nguyen, Smith, Peay, and Kennedy (2015) and was generally caused by the usually poorer quality of reverse sequencing reads of the Illumina MiSeq platform. The problem of missing extremely rare species, however, is not method specific, as the case of *Kuehneola uredinis* demonstrates. This rare species had a total of 47 sequences in the Illumina data and was initially present as a single sequence in the raw data of the clone libraries. Because singletons got discarded regardless of the method, *Kuehneola uredinis* got discarded from the clone data. The fact that the cloning primer pair had a perfect match to *Kuehneola uredinis* and that this species got picked up once clearly shows that the detection of rare species does not rely on the applied method but rather on sequencing depth and bioinformatic assumptions. Picking a greater number of clones would probably have resulted in at least another sequence of *Kuehneola uredines*, and hence detection of this species. Despite failing to detect two rare species by some methods, other rare species, such as *Uromyces dactylidis* and *Puccinia hordei*, could be detected regardless of the method.

Another way of easily missing species when merging paired‐end sequencing reads is to lose “too long” sequences, since these would not overlap. This can be simply tested by not merging the reads and using forward and reverse reads separately. In this study, we found no rust fungus species getting lost during merging as a result of “too long” sequences. The actual Illumina sequencing process, however, is known for discriminating against longer amplicons (Allen et al., 2016). Although less likely than, for instance, a primer mismatch, the *Puccinia* species that could not be detected by NGS metabarcoding but could by cloning could possibly have been missed during the next‐generation sequencing process due to slightly longer amplicons. We did not compare abundance data to a field survey or biomass, but found no significant difference in relative abundances of OTUs on plot level among NGS metabarcoding and cloning. This suggests that any biases in quantification using molecular techniques are not method dependent. Despite issues arising from PCR (yet common for most molecular methods) such as the difference in rRNA copy numbers, several studies do show NGS metabarcoding to be successful for semiquantitative abundance estimation of, for example, feather mite communities in birds (Diaz‐Real, Serrano, Piriz, & Jovani, 2015), fish and amphibians in freshwater ecosystems (Evans et al., 2016), plant–pollinator interactions (Pornon et al., 2016), the biomass of macroinvertebrates (Elbrecht & Leese, 2015), and fungi (Taylor et al., 2016). These studies suggest that if obstacles associated with PCR biases can be overcome, NGS metabarcoding holds promising potential not only for the detection but also for the quantification of species. Moreover, PCR‐free techniques may remedy primer and amplification biases (including chimera formation) in the near future. Different gene copy numbers still pose a significant challenge for biomass estimates but could be overcome with the growing number of whole genome databases.

Next‐generation sequencing metabarcoding has been increasingly recognized as a promising tool for biomonitoring species and complex communities at large scales (Holdaway et al., 2017). In recent cases, it has been applied to plant pathogenic fungi (Merges et al., 2018) and oomycetes (Burgess et al., 2017). It is important to understand the advantages and disadvantages of using NGS metabarcoding for detecting and monitoring important functional groups at the ecosystems scale. Our study suggests that rust fungi can be tracked from within a larger NGS metabarcoding dataset, which should facilitate the future monitoring of this critically important group of fungi.

## CONFLICT OF INTEREST

The authors declare that the research was conducted in the absence of any commercial or financial relationships that could be construed as a potential conflict of interest.

## AUTHORS CONTRIBUTION

The samples used in this paper were part of a study led by RH, IAD, JRW, and KHO. JRW, IAD, RH, and KHO conceived primary funding, with additional funding from the Bio‐Protection Research Centre, led by TRG. IAD and TRG advised AM. AM collected the samples with help from RH and others. AM, CKL, and JRW developed the methods, and AM and CKL carried out molecular characterization. AM and IAD conducted data analysis. AM produced the figures and wrote the first draft. All other authors provided editorial input.

## ETHICS STATEMENT

This article does not contain any studies with human participants or animals performed by any of the authors.

## Data Availability

The data associated with the paper are available from the Manaaki Whenua data repository at https://doi.org/10.25898/KK41-CY40.
