# Supplementary material for: Biases in the metabarcoding of plant pathogens using rust fungi as a model system
Source: Microbiologyopen. 2018 Dec 25;8(7):e00780. doi: 10.1002/mbo3.780 (PMC6612544; doi:10.1002/mbo3.780)
Supplement: Supplementary file 1 [file MBO3-8-e00780-s001.docx]

Figure S1. Map of the 30 grassland study sites across New Zealand. Plot location details are stored in New Zealand’s National Vegetation Survey repository (NVS), https://nvs.landcareresearch.co.nz/ (accessed 1 January 2018).

Figure S2. Neighbour-net phylogeny of dominant (upper 50% of rank abundance) rust fungal operational taxonomic units (OTUs) detected by three methods: Illumina (squares), Ion Torrent (circles), cloning (triangles).

Figure S3. Network representing shared and unique dominant (upper 50% of rank abundance) rust fungal operational taxonomic units (OTUs) between the three methods. Edge width represents proportional abundance of an OTU within method. Species identities are the best BLAST match. OTUs found in each method are considered to be the same >98.5% sequence similarity.

Figure S4. Neighbour-net phylogeny of rare (lower 50% of rank abundance) rust fungal operational taxonomic units (OTUs) detected by methods: Illumina (squares), Ion Torrent (circles), cloning (triangles).

Figure S5. Network representing shared and unique rare (lower 50% of rank abundance) rust fungal operational taxonomic units (OTUs) between methods. Edge width represents proportional abundance of an OTU within method. Species identities are the best BLAST match. OTUs found in each method are considered to be the same >98.5% sequence similarity.

Table S1. Basic sequence information of the three methods. Clusters/OTUs include non-target organisms.

| Method | Raw sequences | Quality filtered sequences | Clusters/OTUs |
| --- | --- | --- | --- |
| Illumina MiSeq | 9793304 | 6216652 | 2347 |
| Ion Torrent PGM | 8090551 | 4946445 | 2947 |
| Cloning + Sanger sequencing | 1387 | 1362 | 42 |

Figure S6. Standard diagnostic plots (result of the gam.check() function) of the model describing relative abundances, methods and OTUs.
